# Predictors of tunneling time in transoral robotic thyroidectomy: impact of anatomical measurements

**DOI:** 10.1007/s00405-026-10526-z

**Published:** 2026-08-19

**Authors:** Minwoo Kim, Seo Young Kim, Pilkeun Jang, Jeong-Yeon Ji, Wonjae Cha, Kyung Tae, Woo-Jin Jeong

**Affiliations:** 1 Department of Otorhinolaryngology-Head and Neck Surgery, Seoul National University Bundang Hospital, Seoul National University College of Medicine, 300 Gumi-dong, Bundang-gu, Seongnam, 13620 Korea; 2 Department of Otorhinolaryngology-Head and Neck Surgery, Chung-Ang University Hospital, Chung-Ang University College of Medicine, Seoul, Korea; 3 Department of Otorhinolaryngology-Head and Neck Surgery, College of Medicine, Hanyang University, Seoul, Republic of Korea

**Keywords:** Transoral robotic thyroidectomy, Tunneling time, Anatomical measurements, Skin-to-thyroid cartilage notch thickness, Mentum-to-thyroid cartilage notch distance

## Abstract

**Purpose:**

To identify anatomical and clinical predictors of tunneling time during transoral robotic thyroidectomy via the vestibular approach (TORTVA) and to clarify the potential utility of body habitus-related predictors for case selection.

**Methods:**

We retrospectively reviewed 33 consecutive patients who underwent TORTVA between October 2023 and December 2024. Tunneling time was defined as the interval from vestibular incision to completion of the subplatysmal tunnel. Preoperative sagittal neck computed tomography (CT) images were used to measure linear distances and angles between the mentum, hyoid bone, thyroid cartilage, thyroid isthmus, and sternal notch, as well as anterior neck soft tissue thickness. Clinicopathologic data and CT measurements were analyzed using univariate and multivariate linear regression.

**Results:**

The mean tunneling time was 25.3 min, with no significant difference observed between males (25.6 min) and females (25.2 min). In multivariate linear regression analysis, the skin-to-thyroid cartilage notch thickness (tST) (B=0.550, *p*=.024) and the mentum-to-thyroid cartilage notch distance (dMT) (B=0.347, *p*=.028) were significantly associated with tunneling time (*p*=.006).

**Conclusion:**

Tunneling time was associated with skin thickness over the thyroid cartilage notch and the mentum-to–thyroid cartilage notch distance. These exploratory findings may inform future studies on patient selection and technical difficulty in TORTVA, particularly for surgeons early in the learning curve.

**Supplementary Information:**

The online version contains supplementary material available at https://doi.org/10.1007/s00405-026-10526-z.

## Introduction

The field of thyroid surgery has undergone transformative advancements over the past two decades, driven by the development of robotic techniques aimed at improving cosmetic outcomes and reducing patient morbidity. Since the transoral endoscopic thyroidectomy vestibular approach (TOETVA) was firstly introduced in 2015, it has been rapidly developed and widely adopted for the treatment of thyroid cancer [1]. With the integration of robotic technology into thyroidectomy, transoral robotic thyroidectomy via the vestibular approach (TORTVA) has emerged to complement the limitation of TOETVA, such as two-dimensional visualization and rigid instrumentation [2, 3, 4].

Tunneling, the initial step in TORTVA, involves creating a subcutaneous working space between the oral vestibule and the thyroid region [5]. This process is technically demanding and time-intensive, often influenced by patient-specific anatomical factors such as skin thickness, mandibular protrusion, and neck length. Despite its importance, existing literature on robotic or endoscopic thyroidectomy has predominantly focused on overall operation time or specific complications, leaving tunneling time largely unexplored [4, 6–9]. Studies on TOETVA have identified factors such as body mass index (BMI) and mandibular jawline as being unrelated with operation time [7–9, 10], while lobe size has been found to be a significant predictor of prolonged operation time [8].

This study aims to evaluate factors that influence tunneling time in TORTVA using preoperative imaging data. To our knowledge, few studies have specifically examined anatomical factors associated with tunneling time in TORTVA. Identifying anatomical features that correlate with prolonged tunneling time during preoperative evaluation can help surgeons anticipate technical challenges and refine patient selection criteria.

## Patients and Methods

### Patients

This retrospective analysis was approved by the Institutional Review Board(IRB No. B-2501-951-107). Patients who underwent TORTVA between October 2023 and December 2024 were included in the analysis. Preoperative neck CT scans were performed for all patient in the supine position, with the neck carefully maintained in a neutral position using a headrest cushion to avoid flexion or extension. Patients with a history of previous neck surgery or radiation, congenital neck abnormalities such as thyroglossal duct cysts or spinal conditions including ankylosing spondylitis, herniated disc and scoliosis were excluded from the study. All surgeries were performed by a single surgeon, and all preoperative imaging measurements were conducted by a single author.

### CT image analysis

**Fig. 1:**
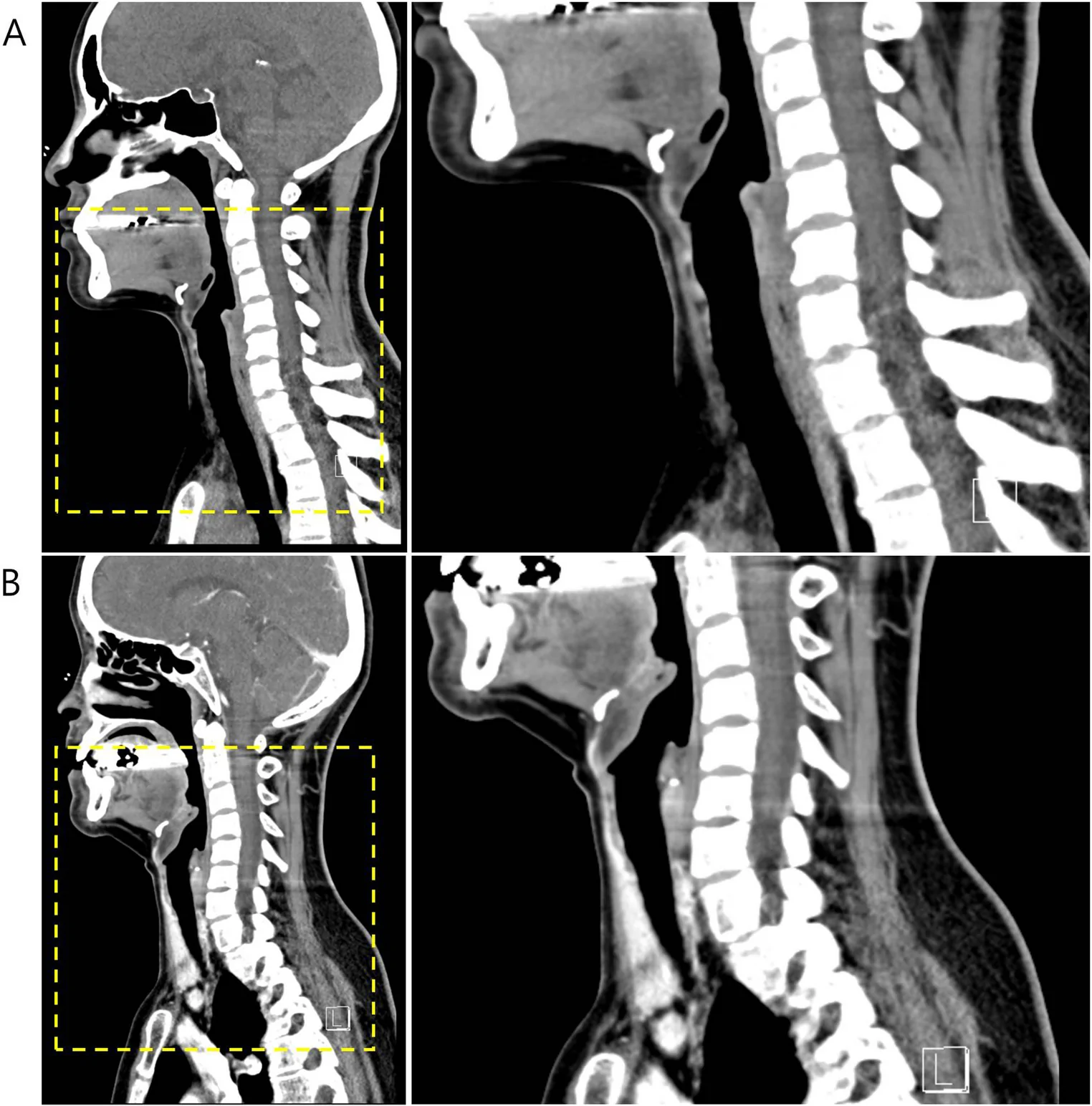
Sagittal CT images obtained using a 250% zoom, a window level of 45, and a window width of 300. (**A**, **B**) Representative images from two different patients demonstrating the same standardized CT viewing settings

All CT measurements were taken from the sagittal sections using the INFINITT PACS Viewer (INFINITT Healthcare Co., Ltd., Seoul, Korea). In the axial view, the midline at the thyroid cartilage notch level was selected as the reference line, and the corresponding sagittal view was observed. All measurements were obtained using CT settings of 250% zoom, a window level of 45 and a window width of 300 (Fig. 1).

**Fig. 2:**
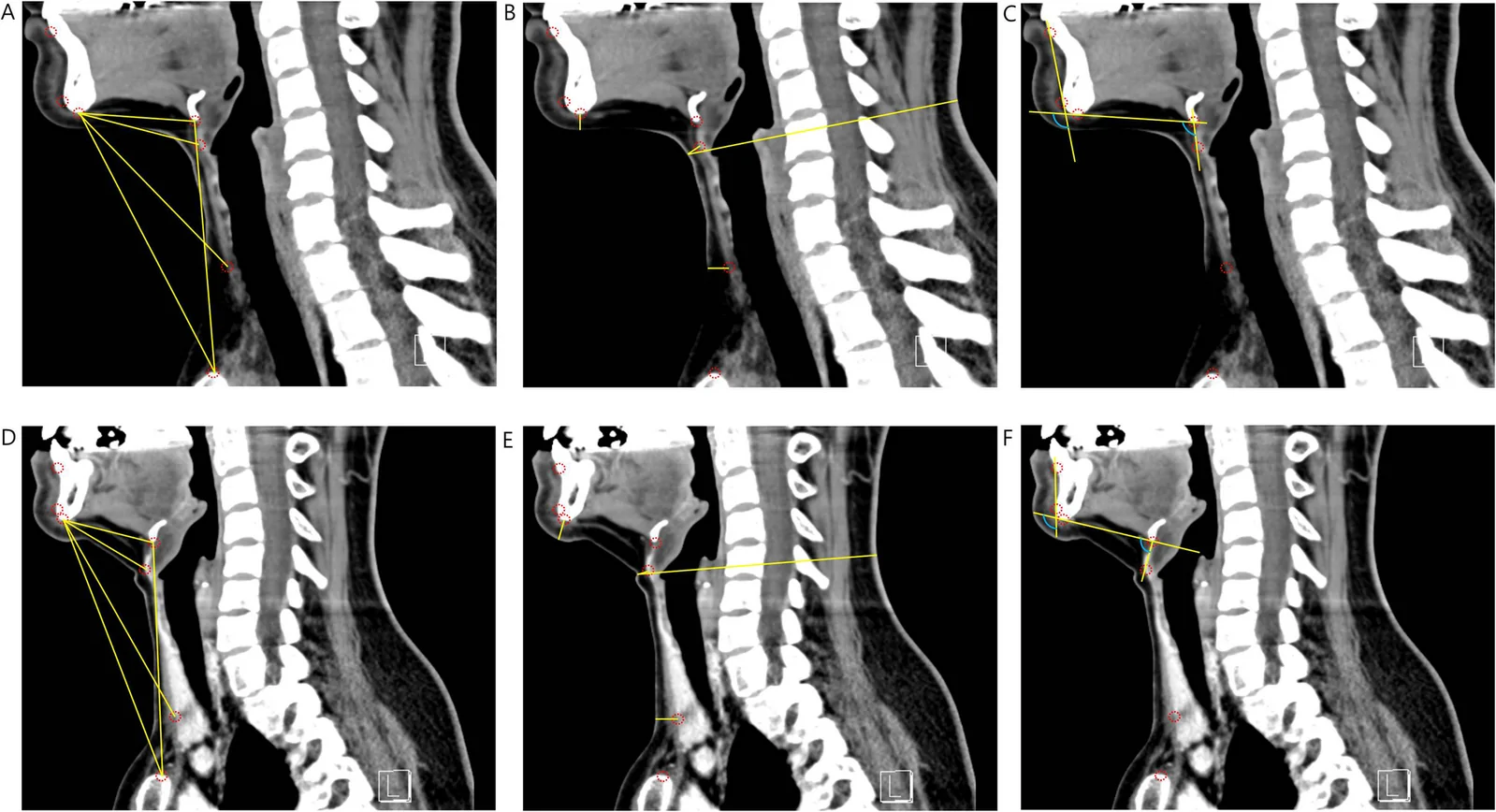
Sagittal CT images illustrating the measurement of tunneling-related anatomical parameters in the two representative patients shown in Fig. 1. (**A**–**C**) Measurements in the first patient. (**D**–**F**) Measurements in the second patient. The red circles indicate the predefined anatomical landmarks described in Supplementary Table 1. (**A**, **B**, **D**, **E**) Linear distance measurements based on these anatomical landmarks. (**C**, **F**) Angular measurements based on the corresponding anatomical landmarks

Seven anatomical points were initially identified and marked to facilitate serial measurements, resulting in a total of 11 measurements (Fig. 2). Detailed descriptions of these anatomical points and measurements are provided in Supplementary Table 1. The thyroid cartilage notch and the inferior midline of the thyroid isthmus were identified and marked using a 3D cursor function, referencing both axial and coronal sections to accurately locate and project the point onto the sagittal sections. Thyroid volumes were measured using CT by applying the volumetric ellipsoid formula (height x width x depth x correction factor 0.529) [11]. The height of each thyroid lobe was measured in coronal sections, while the width and depth were measured in axial sections. The volume of each lobe was calculated individually, and the total thyroid volume was determined by summing the volumes of both lobes.

All CT measurements were performed by a single observer using predefined anatomical landmarks and standardized measurement definitions. The observer was blinded to tunneling time during measurement. To assess intra-rater reliability, measurements of tST and dMT were repeated in all 33 patients by the same observer while blinded to the initial measurements.

### Tunneling time

All procedures were performed by a single experienced surgeon. Tunneling time was defined as the time required from the initial incisions in the oral vestibule to placement of three trocars. Tunneling time was obtained from standardized operative records documented immediately after each procedure, using predefined criteria, and the same definition was applied consistently across all cases. Three incisions were made: one midline incision (1 cm) located 1 cm below the inner lip border to optimize mobility, and two bilateral incisions (0.5 cm) positioned 1 cm medial to both labial commissures. Hydrodissection was performed using 20–30 mL of an epinephrine solution (1:200,000) through the incisions down to the subplatysmal plane to facilitate dissection. Skin flap elevation was achieved through blunt dissection using a sequential approach with curved Halsted mosquito hemostatic forceps, curved Crile hemostatic forceps, curved Kelly hemostatic forceps, curved Wertheim atraumatic parametrium clamp and 8-mm-tipped vascular surgical tunneler. The dissection extended from lip to the sternal notch. The same instruments and technique were used for blunt dissection through the bilateral incisions. Following dissection, three 10-mm trocars were inserted through the incisions. The midline trocar was maneuvered to interconnect with each lateral trocar, enabling the creation of a continuous working space in a blind manner from the thyroid cartilage to the sternal notch.

### Data analysis

Preoperative evaluation included age, sex, medical and surgical history, general physical measurements (weight, height, and BMI), and surgical time variables, including tunneling time, console time, and total operation time.

Statistical analyses were performed using IBM SPSS Statistics version 27 (IBM, Armonk, NY). Group comparisons of continuous variables were conducted using the Mann–Whitney U test. Correlation analyses were performed using Pearson’s correlation coefficient, with Spearman’s rank correlation coefficient applied for subgroup analyses as appropriate. Multivariable linear regression was used to evaluate independent factors associated with tunneling time. Stepwise selection was used as an exploratory tool to reduce the number of candidate predictors in the context of a limited sample size. A *p*-value < 0.05 was considered statistically significant. Intraclass correlation coefficients (ICCs) with 95% confidence intervals were calculated using a two-way mixed-effects model with absolute agreement.

Given the limited sample size (*n* = 33), a parsimonious modeling strategy was applied to reduce the risk of overfitting. Candidate variables were selected based on a priori clinical relevance to the tunneling pathway. Stepwise selection was used as an exploratory approach to derive a simplified multivariable model. To minimize potential multicollinearity, only clinically relevant, non-redundant variables were included in the multivariable analysis.

## Results

The patient’s clinicopathologic characteristics were summarized in Table 1. Between October 2023 and December 2024, a total of 33 patients underwent TORTVA at our hospital. Of these, 11 patients (33.3%) were male, and 22 patients (66.7%) were female. The mean age was 42.3 ± 11.5 years, and the mean tunneling time was 25.3 ± 8.2 min. Lobectomy only was performed in 54.5% of cases (*n* = 18), total thyroidectomy only was performed in 15.2% (*n* = 5), lobectomy with central neck dissection (CND) was 6.1% (*n* = 2), total thyroidectomy with CND was 3% (*n* = 1), and Isthmusectomy only in 21.2% (*n* = 7). Among the diagnoses, 30 patients had papillary thyroid carcinoma, while 1 patient was diagnosed with follicular thyroid carcinoma, 1 with follicular adenoma, and 1 with oncocytic adenoma.

**Table 1:** Patient demographics

	Patients (*n* = 33)
Age (yrs), mean ± SD	42.3 ± 11.5
Sex (%)	
Male	11 (33.3)
Female	22 (66.7)
Height (cm), mean ± SD	163.9 ± 7.8
Weight (kg), mean ± SD	66.3 ± 14.9
BMI (kg/m^2^), mean ± SD	24.5 ± 4.3
HTN (%)	3 (9.1)
DM (%)	1 (3.0)
Cervical spine disease (%)	0 (0.0)
Thyroiditis (%)	2 (6.1)
Diagnosis (%)	
Papillary thyroid carcinoma	30 (91.0)
Follicular thyroid carcinoma	1 (3.0)
Follicular adenoma	1 (3.0)
Oncocytic adenoma	1 (3.0)
Operation (%)	
Total thyroidectomy only	5 (15.2)
Total thyroidectomy with bilateral CND	1 (3.0)
Unilateral lobectomy only	18 (54.5)
Unilateral lobectomy with unilateral CND	2 (6.1)
Isthmusectomy	7 (21.2)
Tunneling time (min), mean ± SD	25.3 ± 8.2

*BMI *Body mass index, *HTN* Hypertension, *DM* Diabetes mellitus, *CND* Central neck dissection

Table 2 presents the results of factors potentially influencing tunneling time, including CT measurements, and compares these results according to sex. The mean tunneling time was 25.6 ± 8.1 min in males and 25.2 ± 8.4 min in females, with no significant difference observed (*p*=.925). Additionally, no significant differences were observed between males and females for mentum-to-hyoid bone distance (dMH), mentum-to-thyroid cartilage notch distance (dMT), hyoid-to-sternal notch distance (dHS), skin-to-mentum thickness (tSM), skin-to-thyroid cartilage notch thickness (tST), mentum-to-hyoid bone-to-thyroid cartilage notch angle (aMHT), or thyroid size (Table 2).

**Table 2:** Comparison of patient’s results according to sex

	Total (*n* = 33)	Male (*n* = 11)	Female (*n* = 22)	*p*-value
Age (yrs)	42.3 ± 11.5	40.0 ± 8.1	43.4 ± 12.9	0.462
Height (cm)	163.9 ± 7.8	172.5 ± 4.4	159.6 ± 5.0	< 0.001^*^
Weight (kg)	66.3 ± 14.9	78.2 ± 16.3	60.3 ± 10.1	< 0.001^*^
BMI (kg/m^2^)	24.5 ± 4.3	26.2 ± 5.1	23.6 ± 3.6	0.044^*^
Tunneling time (min)	25.3 ± 8.2	25.6 ± 8.1	25.2 ± 8.4	0.925
dMH (mm)	41.3 ± 6.8	41.9 ± 9.4	40.9 ± 5.2	0.866
dMT (mm)	43.4 ± 8.3	46.1 ± 9.9	41.3 ± 6.4	0.201
dMI (mm)	82.9 ± 14.1	94.6 ± 11.7	76.8 ± 11.5	0.001^*^
dMS (mm)	104.9 ± 18.8	116.8 ± 17.9	97.6 ± 15.3	0.005^*^
dHS (mm)	97.2 ± 12.6	97.9 ± 18.5	96.4 ± 8.9	0.462
tSM (mm)	6.7 ± 2.1	7.3 ± 1.9	6.3 ± 2.1	0.468
tST (mm)	8.7 ± 5.3	5.6 ± 5.4	10.2 ± 4.8	0.178
tSI (mm)	10.6 ± 6.4	12.4 ± 9.2	9.8 ± 4.7	0.002^*^
ND (mm)	116.4 ± 14.0	132.2 ± 8.7	108.2 ± 8.4	< 0.001^*^
aAMH (^o^)	103.2 ± 13.0	112.5 ± 12.2	97.9 ± 10.6	0.002^*^
aMHT (^o^)	86.9 ± 23.3	93.6 ± 25.5	81.8 ± 20.4	0.281
Thyroid size (cm^3^)	17.1 ± 4.9	17.7 ± 3.4	17.0 ± 5.6	0.534

*dMH* Mentum-to-hyoid bone distance, *dMT* Mentum-to-thyroid cartilage notch distance, *dMI* Mentum-to-thyroid isthmus distance, *dMS* Mentum-to-sternal notch distance, *dHS* Hyoid bone-to-sternal notch distance, *tSM* Skin-to-mentum thickness, tST Skin-to-thyroid cartilage notch thickness, *tSI* Skin-to-thyroid isthmus thickness, *ND* Neck anterior to posterior diameter, *aAMH* Alveolar ridge-to-mentum-to-hyoid bone angle, *aMHT* Mentum-to-hyoid bone-to-thyroid cartilage notch angle

^*^: *p*<.05, All records above were mean ± SD

A total of 16 anatomical and clinical variables were examined in univariate analyses to explore potential associations with tunneling time. On univariate analysis, weight, BMI, dMH, dMT, dHS, tST, tSI and aMHT were significantly associated with prolonged tunneling time (Table 3).

**Table 3:** Comparison of patient’s results with Tunneling time

		Univariate analysis		Multivariate analysis	
	Results	coefficient	*p*-value	Standardized B (95% CI)	*p*-value
Age (yrs)	42.3 ± 11.5	-0.293	0.474		
Height (cm)	163.9 ± 7.8	0.114	0.260		
Weight (kg)	66.3 ± 14.9	0.357	0.019^*^		
BMI (kg/m^2^)	24.5 ± 4.3	0.375	0.015^*^		
dMH (mm)	41.3 ± 6.8	0.392	0.011^*^		
dMT (mm)	43.4 ± 8.3	0.389	0.011^*^	0.347 (0.040–0.654)	0.028^*^
dMI (mm)	82.9 ± 14.1	-0.226	0.100		
dMS (mm)	104.9 ± 18.8	-0.116	0.257		
dHS (mm)	97.2 ± 12.6	-0.363	0.017^*^		
tSM (mm)	6.7 ± 2.1	0.133	0.227		
tST (mm)	8.7 ± 5.3	0.397	0.010^*^	0.550 (0.076–1.025)	0.024^*^
tSI (mm)	10.6 ± 6.4	0.352	0.021^*^		
ND (mm)	116.4 ± 14.0	0.197	0.132		
aAMH (^o^)	103.2 ± 13.0	0.031	0.432		
aMHT (^o^)	86.9 ± 23.3	0.343	0.024^*^		
Thyroid size (cm^3^)	17.1 ± 4.9	-0.029	0.436		

*dMH* Mentum-to-hyoid bone distance, *dMT* Mentum-to-thyroid cartilage notch distance, *dMI* Mentum-to-thyroid isthmus distance, *dMS* Mentum-to-sternal notch distance, *dHS* Hyoid bone-to-sternal notch distance, *tSM* Skin-to-mentum thickness, *tST* Skin-to-thyroid cartilage notch thickness, *tSI* Skin-to-thyroid isthmus thickness, *ND* Neck anterior to posterior diameter, *aAMH* Alveolar ridge-to-mentum-to-hyoid bone angle, *aMHT* Mentum-to-hyoid bone-to-thyroid cartilage notch angle, *CI* Confidence interval

^*^: *p*<.05, All records above were mean ± SD

Multivariate linear regression analysis was performed to identify factors influencing tunneling time among the 33 patients. To improve model stability and reduce the risk of overfitting, the multivariable model was intentionally restricted to a limited number of clinically relevant, non-redundant predictors. Variables that showed no significant sex differences (dMH, dMT, dHS, tSM, tST, aMHT, and thyroid size) were included in the model. Using a stepwise selection method, the final model yielded an *R*^2^ of 0.281 with *F* = 6.064 (*p*=.006). The analysis identified tST (*B*=0.550, SE = 0.233, 95% CI: [0.076, 1.025], *p*=.024) and dMT (*B*=0.347, SE = 0.150, 95% CI: [0.040, 0.654], *p*=.028) as significant predictors of tunneling time (Table 3). The constant was estimated at 5.638. Given the modest explanatory power of the model, these associations should be interpreted with caution. Intra-rater reliability was excellent for both key CT-derived measurements. The ICC was 0.986 (95% CI, 0.973–0.993) for tST and 0.995 (95% CI, 0.989–0.997) for dMT.

To evaluate the correlation between tunneling time and operation time, analysis was conducted on 18 patients who underwent lobectomy only. Tunneling time was defined as the interval from vestibular incision to completion of subplatysmal tunnel creation. Console time was defined as the duration from robotic docking to undocking. Total operation time was defined as the interval from skin incision to skin closure. The mean tunneling time was 25.6 ± 9.9 min, console time was 139.8 ± 35.7 min, and operation time was 188.4 ± 32.5 min. When comparing these times by sex using the Mann-Whitney U test, no significant differences were observed for tunneling time (*p*=.982), console time (*p*=.551), or operation time (*p*=.677). Spearman’s rank correlation analysis revealed no significant correlation between tunneling time and console time (*p*=.159) or between tunneling time and operation time (*p*=.849). However, a significant positive correlation was observed between console time and operation time (*p*<.001).

## Discussion

This study provides novel insights into the factors influencing tunneling time during transoral robotic thyroidectomy via the vestibular approach (TORTVA). Tunneling, a technically demanding and time-intensive step, involves creating a subcutaneous working space between the oral vestibule and the thyroid region prior to robotic docking in TORTVA [4–5]. This process is performed blindly, relying on the surgeon’s tactile feedback and anatomical knowledge to safely and effectively establish the working space. Proper creation of this space is crucial, as it directly affects the feasibility and safety of subsequent surgical steps, including robotic docking and dissection. Inadequately or improperly formed spaces can lead to complications, hinder visualization, and compromise the overall success of the procedure [12]. Therefore, tunneling plays a pivotal role in ensuring the smooth progression and ultimate success of TORTVA. In this study, all cases were performed with a consistent flap elevation range, and the included cases were conducted after the surgeon had surpassed the early learning curve phase to minimize variability related to surgical experience.

Previous research has investigated operation times for robotic thyroidectomy or TOETVA [6–9], however none have focused on this specific phase of the procedure or its unique challenges. The findings of this study aim to address this gap by providing valuable insights into an underexplored aspect of robotic thyroidectomy that has significant implications for surgical efficiency and patient outcomes. As the first study to systematically analyze these factors, it highlights the importance of objective anatomical assessment in predicting tunneling time and optimizing patient selection. Importantly, our intent is not to advocate obtaining CT solely to predict tunneling difficulty; rather, when preoperative imaging is already available for standard indications, CT-based measurements provide a reproducible, quantitative framework that helps explain and formalize what surgeons often appreciate clinically. By identifying key anatomical predictors such as skin-to-thyroid cartilage notch thickness (tST) and mentum-to-thyroid cartilage notch distance (dMT), this study offers practical guidance for surgeons, particularly those new to TORTVA. These findings can assist surgeons in anticipating technical challenges, refining patient selection criteria, and improving surgical outcomes. Moreover, the two independent predictors identified here (tST and dMT) conceptually correspond to readily appreciable elements of patient body habitus on routine preoperative assessment—such as subcutaneous tissue thickness over the anterior neck and the effective “chin-to-laryngeal” working distance—allowing surgeons to incorporate these insights even without formal CT-based measurement.

In this study of 33 patients, the mean tunneling time was 25.3 min, with no significant difference between males and females (*p*=.925) (Table 2). Since no prior research has specifically analyzed factors influencing tunneling time, anatomical variables along the tunneling route were carefully selected for evaluation. These included measurements of length, thickness, and angle based on preoperative CT imaging. To minimize potential bias caused by sex differences, factors such as height, weight, BMI, dMI, dMS, tSI, ND, and aAMH—where significant differences between males and females were observed—were excluded from the analysis (Table 2). This adjustment ensured that the identified predictors of tunneling time were independent of sex-related variations.

Univariate analysis revealed that several factors were significantly associated with tunneling time regardless of sex. Among these were the distance from the mentum to the hyoid bone (dMH), the distance from the mentum to the thyroid cartilage notch (dMT), the distance from the hyoid bone to the sternal notch (dHS), the thickness of the skin at the thyroid cartilage notch (tST), and the mentum-to-hyoid-to-thyroid cartilage notch angle (aMHT). Specifically, longer distances from the mentum to key anatomical landmarks (dMH and dMT) were associated with prolonged tunneling times, likely due to increased complexity in creating a working space over a larger area. Similarly, greater skin thickness at the thyroid cartilage notch (tST) was associated with more challenging tunneling, as thicker tissue layers may impede efficient dissection. These findings align with intraoperative observations made by surgeons during TORTVA procedures. Interestingly, a larger mentum-to-hyoid-to-thyroid cartilage notch angle (aMHT) was also associated with longer tunneling times. This may reflect variations in neck extension during surgery; however, further studies are needed to confirm this relationship.

Multivariate linear regression analysis identified tST and dMT as independent predictors of tunneling time (Table 3). Although tST and dMT were identified as independent predictors in multivariable analysis, the overall explanatory power of the model was modest (R² =0.28). Therefore, these findings should be regarded as exploratory and hypothesis-generating rather than definitive predictors of tunneling difficulty. Nevertheless, these results indicate that both the distance from the mentum to the thyroid cartilage notch and the thickness of the skin at the thyroid cartilage notch are critical factors influencing tunneling efficiency. From a practical standpoint, while multiple candidate measurements were explored to characterize the tunneling pathway, our results support focusing on these two key parameters as the most informative and clinically interpretable predictors. This highlights the importance of careful attention during tunneling as it progresses through these anatomical regions.

Initially, it was hypothesized that sex, weight, and BMI might play significant roles in influencing tunneling time due to their established associations with surgical outcomes. While these factors showed significance in univariate analysis (Table 3), multivariate regression revealed that their effects were ultimately subsumed by tST and dMT. This finding suggests that while demographic factors may indirectly correlate with tunneling difficulty, they are not independent predictors when anatomical measurements are taken into account. Clinically, however, surgeons should remain aware of sex, weight, and BMI during preoperative evaluations as they may provide preliminary insights into potential challenges. Nonetheless, this study emphasizes that direct measurements from sagittal CT views—specifically tST and dMT—offer a more precise and objective basis for predicting tunneling complexity. At the same time, these CT-derived predictors may be viewed as objective surrogates for “body habitus” features that experienced surgeons often recognize on examination (e.g., thicker anterior neck soft tissue and a longer working distance from the chin), thereby helping to standardize preoperative assessment and communication, particularly in training settings. An important conceptual consideration is whether the CT-derived parameters identified in this study represent simple surrogates of general body habitus or reflect independent anatomical constraints relevant to the tunneling pathway. In this study, tST and dMT were not intended as substitutes for global body habitus measures such as BMI or weight. Rather, they were designed to capture localized, region-specific anatomical characteristics—namely anterior neck soft-tissue thickness and the effective chin-to-laryngeal working distance—that may not be adequately reflected by general anthropometric indices. And, factors such as aAMH—initially hypothesized to correlate with tunneling difficulty due to mentum protrusion [10, 13]—did not show any significant association with tunneling time (*p*=.432) (Table 3). Xing Yu et al. (2022) compared surgical outcomes in TOETVA using jawline classifications but did not report any findings specifically related to tunneling and concluded that the surgery achieved safe and effective outcomes regardless of jawline [9]. Additionally, thyroid size was analyzed as a potential factor influencing tunneling time based on its established role in operation time for TOETVA [8, 11]. However, no significant relationship was found between thyroid size and tunneling time in this study (*p*=.436) (Table 3).

To assess the clinical relevance of tunneling time in relation to overall operation duration, a subgroup analysis was performed in 18 patients who underwent lobectomy only, as total operation time may vary depending on the extent of surgery, including total thyroidectomy or central neck dissection [6, 8]. The mean tunneling time in this subset was 25.6 ± 9.9 min; however, tunneling time showed no significant correlation with console time (139.8 ± 35.7 min, *p* = .159) or total operation time (188.4 ± 32.5 min, *p* = .849). In contrast, console time was significantly correlated with total operation time (*p* < .001), suggesting that console time accounts for a larger proportion of overall operation time than tunneling time in TORTVA. Additionally, non-surgical components such as robotic docking and undocking times or instrument switching on robotic arms may contribute significantly to total operation duration in TORTVA [14] Importantly, the clinical relevance of predicting tunneling time should not be interpreted solely in terms of reducing total operation time. Tunneling represents a technically demanding, blind step that determines the quality of the working space prior to robotic docking. Prolonged or difficult tunneling may disrupt surgical workflow, necessitate corrective maneuvers such as re-tunneling or undocking, and increase technical stress, even if total operation time is not ultimately prolonged. Although prolonged tunneling did not translate into longer operation time in this experienced cohort, anticipating tunneling difficulty may still be clinically meaningful, particularly for surgeons early in the learning curve. For novice or beginner surgeons, challenging tunneling may complicate early case selection, increase procedural stress, and potentially compromise safety during the initial adoption phase. Accordingly, the present findings should be interpreted as exploratory and focused on technical workflow considerations rather than direct optimization of operative duration or postoperative outcomes.

This study has several limitations that should be considered when interpreting its results. First, as a single-center retrospective analysis with a relatively small sample size, the generalizability of these findings may be limited. In addition, the limited sample size may increase the risk of model instability and overfitting in multivariable analyses. External or internal validation was not performed. Therefore, the findings should be interpreted as exploratory and require validation in larger, independent cohorts. Larger multicenter prospective studies are required to confirm these findings. Second, although intra-rater reliability was assessed and demonstrated excellent reproducibility for the two measurements included in the final model, inter-observer reliability was not evaluated. Therefore, the generalizability of these measurements across different observers remains uncertain. Although predefined anatomical landmarks and standardized measurement definitions were applied, measurement-related variability cannot be excluded. Future studies should include formal reliability testing across multiple observers. Additionally, preoperative CT imaging was performed with patients in a neutral neck position, which may differ from the extended neck position used during surgery. This difference may alter the spatial relationships between anatomical structures and affect measurements along the tunneling pathway. Therefore, these findings should be interpreted with caution. Prospective validation using standardized imaging protocols or surgical positioning is required. Another limitation lies in the inclusion of non-surgical components within total operation time measurement. Factors such as robotic docking and undocking times or instrument switching on robotic arms are relevant to overall surgical efficiency but do not directly reflect technical aspects of tunneling. In addition, tunneling-related intraoperative events (e.g., bleeding, workflow interruption, or need for re-tunneling) were not systematically collected, and their relationship with tunneling time could not be evaluated. A more detailed analysis isolating surgical steps from logistical factors could provide a clearer understanding of how tunneling time contributes to overall operation time. Finally, the modest explanatory power of the final model suggests that additional, unmeasured factors (e.g., tissue quality and surgeon technique) may also influence tunneling time, warranting further investigation.

Despite these limitations, this study provides valuable insights into TORTVA by identifying key anatomical predictors of tunneling time and emphasizing their clinical relevance for preoperative planning. By focusing on patient-specific anatomical features, this research lays important groundwork for improving efficiency and safety in minimally invasive thyroid surgery. These findings are particularly beneficial for surgeons new to TORTVA by offering guidance on patient selection and helping anticipate technical challenges during the procedure. Rather than proposing routine additional imaging, our findings support incorporating simple, body-habitus–informed assessment—supported by objective metrics when imaging is already available—into early case selection and preoperative counseling for surgeons adopting TORTVA. Furthermore, incorporating neck extension into preoperative CT protocols could help establish more accurate predictive models and potentially redefine imaging standards for TORTVA. Future research should also explore additional anatomical or procedural factors that may influence both tunneling and overall operation times to contribute to improved patient outcomes, enhanced training for novice surgeons, and improved surgical efficiency. 

## Supplementary Information

Supplementary Material 1. Supplementary Table 1. Definitions of anatomical landmarks and linear and angular measurements used for sagittal neck CT analysis in transoral robotic thyroidectomy.

## Data Availability

The datasets generated during and/or analyzed during the current study are available from the corresponding author on reasonable request.

## Declarations

### Use of artificial intelligence (AI) tools

The authors did not use artificial intelligence (AI) tools to generate content for this manuscript.
