# Supplementary material for: Predictors of tunneling time in transoral robotic thyroidectomy: impact of anatomical measurements
Source: Eur Arch Otorhinolaryngol. 2026 Aug 19;283(11):7441–9. doi: 10.1007/s00405-026-10526-z (PMC13748044; doi:10.1007/s00405-026-10526-z)
Supplement: Supplementary file 1 [file 405_2026_10526_MOESM1_ESM.docx]

**Supplementary Table 1.**

All anatomical measurements were obtained from sagittal CT images using predefined anatomical landmarks. Distances were measured in millimeters, and angles were measured in degrees.

| Anatomical point name | Description |
| --- | --- |
| The alveolar process ridge | The ridge of the alveolar process in the mandible |
| The anterior mental border | The point where a tangent from the alveolar process ridge meets the most anterior aspect of the mentum |
| The hyoid bone inferior border | The lowest point on the hyoid bone |
| The inferior mental border | The point where a tangent from the inferior border of hyoid bone meets the most inferior aspect of the mentum |
| The thyroid cartilage notch | The superior notch of the thyroid cartilage |
| The inferior midline of the thyroid isthmus | The lowest central point on the thyroid isthmus |
| The sternal notch | The most superior aspect of the sternal notch |
| Measurement name | Description |
| Mentum-to-hyoid distance (dMH) | Distance from the inferior mental border to the hyoid bone inferior border |
| Mentum-to-thyroid cartilage notch distance (dMT) | Distance from the inferior mental border to the thyroid cartilage notch |
| Mentum-to-thyroid isthmus distance (dMI) | Distance from the inferior mental border to the inferior midline of the thyroid isthmus |
| Mentum-to-sternal notch distance (dMS) | Distance from the inferior mental border to the sternal notch |
| Hyoid bone-to-sternal notch distance (dHS) | Distance from the hyoid bone inferior border to the sternal notch |
| Skin-to-mentum thickness (tSM) | Vertical distance from the point of tangency on the inferior mental border to the skin |
| Skin-to-thyroid cartilage notch thickness (tST) | Distance from skin to the thyroid cartilage notch |
| Skin-to-thyroid isthmus thickness (tSI) | Distance from skin to the inferior midline of the thyroid isthmus |
| Neck anterior to posterior diameter (ND) | Neck diameter from skin of the thyroid cartilage notch to posterior neck, parallel to the vertebra |
| Alveolar process ridge-to-mentum-to-hyoid bone angle (aAMH) | Angle of the alveolar process ridge-to-the anterior mental border plane and the inferior mental border-to-the hyoid bone inferior border plane |
| Mentum-to-hyoid bone-to-thyroid cartilage notch angle (aMHT) | Angle of the inferior mental border-to-the hyoid bone inferior border plane and the hyoid bone inferior border-to-the thyroid cartilage notch plane |
